# Women’s perspectives on antenatal breast expression: a cross-sectional survey

**DOI:** 10.1186/s12978-018-0497-4

**Published:** 2018-04-04

**Authors:** Frankie J. Fair, Helen Watson, Rachel Gardner, Hora Soltani

**Affiliations:** 1 Faculty of Health and Wellbeing, Sheffield Hallam University, Collegiate Crescent, Sheffield, UK; 2 Sheffield Maternity Services Liaison Committee and Sheffield user group charity – Forging Families, Sheffield, UK

**Keywords:** Antenatal breast expression, Maternal obesity, Breastfeeding, Survey, Opinions, Compliance

## Abstract

**Background:**

The practice of antenatal breast expression (ABE) has been proposed as a strategy to promote successful breastfeeding. Although there has been some focus on the evaluation of the effects of ABE in promotion of breastfeeding, little or no evidence exists on women’s experiences of ABE or opinions on ABE, particularly amongst overweight or obese women.

**Methods:**

This study aimed to explore women’s knowledge, practices and opinions of ABE, and any differences within the overweight and obese subgroups. A cross-sectional survey was undertaken using an online questionnaire distributed by a maternity user group representative via social media. Quantitative data were analysed using Chi-square and Fisher’s exact tests in SPSS. Simple thematic analysis was used for the qualitative data.

**Results:**

A total of 688 responses were analysed; the sample represented a group of breastfeeding mothers, of whom 64.5% had heard of ABE, 8.2% had been advised to do ABE, and 14.2% had undertaken ABE. Of the women who had been advised to do ABE, 67.9% had complied. Most participants (58.6%) were unsure if ABE was a good idea; however 80.9% would consider doing ABE if it was found to be helpful to prepare for breastfeeding. Women in the overweight or obese subgroups were significantly more likely to have heard of ABE (*p* < 0.001), and positive opinion of ABE also increased with higher BMI groups. The qualitative data demonstrated participants felt ABE may be beneficial when mother or baby have medical problems, and in preparation for breastfeeding, but highlighted their concerns that it may interfere with nature and be harmful, and that they wanted more information and knowledge about ABE.

**Conclusions:**

Amongst women who have breastfed, many have heard of ABE, compliance with advice to undertake ABE is relatively high, and ABE is considered an acceptable practice. Further investigation into the benefits and safety of ABE is warranted, to address the needs of childbearing women for evidence-based information about this practice. If the evidence base is established, overweight and obese pregnant women could be an important target group for this intervention.

**Electronic supplementary material:**

The online version of this article (10.1186/s12978-018-0497-4) contains supplementary material, which is available to authorized users.

## Plain English summary

It has been proposed that the manual expression of breastmilk whilst a woman is still pregnant (ABE) may promote breastfeeding. A few studies have evaluated the effects of ABE on promotion of breastfeeding but to our knowledge no study has focused on exploring women’s experiences and opinions about this practice on a large scale.

This study aimed to investigate women’s experiences of and opinions on this practice by undertaking a questionnaire based survey. This was distributed online and a total of 688 responses were analysed. The respondents were predominately a group of breastfeeding mothers, of whom 64.5% had heard of ABE, 8.2% had been advised to do ABE, and 14.2% had undertaken ABE. Of the women who had been advised to do ABE, 67.9% had followed this advice. Most participants (58.6%) were unsure if ABE was a good idea; however 80.9% would consider doing ABE if it was found to help prepare for breastfeeding. Overweight or obese women were more likely to have heard of ABE, and were equally as positive about undertaking it if it was found to help prepare for breastfeeding. Women felt that ABE may be beneficial when mother or baby have medical problems, and in preparation for breastfeeding, but had concerns that it may interfere with nature and be harmful, and said that they wanted more information about ABE. If further research finds that ABE is safe and effective, it could be developed as an intervention to promote breastfeeding, particularly for overweight and obese pregnant women.

## Background

The World Health Organization recommends that infants are exclusively breastfed until 6 months of age with continued breastfeeding thereafter alongside appropriate complementary foods [1]. The health benefits of breastfeeding for both the mother and the infant are well documented [2–4]. However, the practice of breastfeeding varies extensively and identifying appropriate strategies to promote breastfeeding amongst all women are required.

### Factors influencing breastfeeding

The United Kingdom (UK) Infant feeding Survey has demonstrated that the prevalence of breastfeeding is higher amongst mothers from managerial and professional occupations, those who left education aged over 18, mothers aged 30 and over, mothers from ethnic minority groups and mothers living in the least deprived areas [5].

A lower prevalence of breastfeeding and poorer breastfeeding outcomes among women who are overweight or obese is well supported by epidemiological studies [6–13]. Maternal obesity is associated with up to 13% lower breastfeeding initiation rates, and shorter duration of any breastfeeding and exclusive breastfeeding when compared to women of normal weight [13, 14]. This is of particular concern given the rising rate of overweight and obesity across the globe [15], with rates in women aged 20 years or over within the UK in 2013 standing at 57.2% with a body mass index (BMI) ≥ 25 kg/m^2^ and 25.4% with a BMI ≥30 kg/m^2^ [16].

Although psychosocial factors are significantly associated with the lower incidence of breastfeeding among women with a BMI over 25 kg/m^2^, anatomical and physiological alterations may also play a role. Among these is that women who are overweight or obese may have mammary hypoplasia or insufficient glandular tissue as obesity in childhood negatively affects the development of breast glandular tissue [13]. Many of the characteristics experienced by women who are overweight or obese are consistent with this, including reporting stopping breastfeeding due to perceived insufficient supply [17] and being more likely to try to express in the first 2 months postpartum but less likely to have successfully expressed than women with a normal BMI [18]. A high pre-pregnancy BMI is also a predictor of delayed onset lactogenesis II [19], which is the production of copious milk triggered by progesterone withdrawal after the removal of the placenta [13, 20]. This most critical stage of the lactation cycle [21] is more likely to be delayed (occurring more than 72 h after birth) amongst women with a high BMI than women of normal weight [22]. Various underlying physiological reasons for the increased likelihood of delayed lactogenesis II amongst women with obesity are proposed; the impact of increased oedema [13], increased likelihood of medical problems including gestational diabetes, prolonged labour, caesarean section or preterm birth [6, 23], the role of leptin released from adipose tissue which inhibits oxytocin and milk ejection [13], reduced prolactin response to infant suckling [24], and the impact of insulin imbalance [13].

### Antenatal breast expression

The practice of antenatal breast expression (ABE) has been proposed as a strategy to prevent delayed lactogenesis II in both the academic and consumer literature [25, 26], and as a strategy to overcome the effects of delayed lactogenesis II by ensuring women have a store of expressed milk to prevent the use of formula milk, particularly if the mother has pre-existing or gestational diabetes [27–29]. ABE is widely recommended in UK maternity units [30–43]. A recent survey demonstrated that out of the 56 responding maternity units across 9 geographical regions in the UK, 73% offered ABE to diabetic women, 25% offered ABE to women who had risk factors for neonatal hypoglycaemia, and 19% offered ABE to all women [44]. Furthermore, the practice of ABE is promoted by some lactation support websites [29, 45].

ABE involves expressing colostrum from the breast in the antenatal period, however there is little consensus on timing of onset, frequency, duration and method of expression. Recommendations vary within the academic and consumer literature and local UK guidelines on when to commence ABE. A majority of UK maternity units recommend commencing ABE from 36 to 37 weeks (98%) with only 2% recommending commencing ABE at 35,38 or 39 weeks gestation [44], however consumer literature recommends commencing ABE from 32 to 34 weeks of pregnancy [29]. Similarly recommendations regarding how to express ABE vary, including frequency ranging from once a day [35], building up to 4 times a day [32, 34, 39] a minimum of 4 times a day [43] or as often as the woman wants [36, 40] and duration varying from 5 min [35] up to 20 min at a time [32, 38, 39].

Evidence of the effectiveness or underlying mechanism of action of ABE are still not clear, although several studies including a recent large randomised controlled trial have focused on evaluating the safety and efficacy of ABE among women with diabetes [27, 46–48]. Initial concerns raised in a retrospective cohort study about the safety of ABE in regards to influencing the timing of onset of labour if ABE was commenced prior to 37 weeks’ gestation [49] have not been supported by a large randomised controlled trial of women at low risk of complications with diabetes in pregnancy [48]. This trial found no difference in gestational at birth between those randomised to ABE from 36 weeks’ gestation and women randomised to standard care [48].

Given that ABE is a widely recommended practice in maternity units and on lactation support websites and that limited evaluation of the acceptability of ABE to prepare for breastfeeding has been undertaken, a wide scoping of acceptability and women’s views on this practice merits a focused exploration.

This study was therefore designed to assess the general knowledge of ABE among mothers, their practices surrounding ABE and the acceptability of ABE to them should it be found to be an effective preparation for breastfeeding. It was also aimed to explore any differences in knowledge and acceptability within the overweight and obese subgroup of mothers.

## Methods

A cross-sectional survey using a questionnaire was developed in consultation with maternity user group representatives; individuals representing the opinions of mothers and fathers currently expecting a baby or with a child under 1 year old. The survey questionnaire was generated using the online, cloud-based software, Survey Monkey. Applying a convenience sampling strategy, the questionnaire was distributed from December 2015 to January 2016 through a maternity service user and parenting Facebook group, which was moderated by the maternity user group representative member of the research team. This Facebook group aimed to allow parents to share stories and gain peer support, it was not focussed on method of infant feeding*.*

The questionnaire consisted of a mixture of question types, including free text questions and fixed response options. Demographic data was collected, including place of residence, ethnicity, age, number of pregnancies, and occupation of participants. In order to calculate BMI, the survey included a question about the participants’ height and weight at the time of the survey, in either metric or imperial units.

Survey questions covered the following topics; whether participants had ever breastfed and for how long, whether they had heard of the practice of ABE, had been advised to do ABE and had undertaken ABE, and their opinion on whether ABE was a good idea and if they would consider doing ABE if it was found to be beneficial to breastfeeding.

Ethical approval was obtained from Sheffield Hallam University Research Ethics Committee, study ID: 2015–16/ HWB-HSC-14. Consent was assumed inherent for the participants who completed the questionnaire voluntarily.

### Data analysis

Logical checks and data cleaning were carried out and inconsistencies double checked for clarification. All survey data were double-entered and cleaned using SPSS 21.0. Descriptive statistics including proportions, ranges, means, standard deviation (SD), median and interquartile ranges (IQR) as appropriate were calculated for the demographic data and for closed answer questions. Categorical data were analysed using Chi-square test or Fisher’s exact test where the assumptions for Chi-square test were not satisfied, such as expected count < 5 in over 20% of cells. A *p* value < 0.05 was regarded as indicating statistical significance.

BMI values were calculated from the reported height and weight measurements and grouped into 3 categories; BMI less than 25 kg/m^2^, overweight (BMI of 25–29.9 kg/m^2^) and obese (BMI of 30 kg/m^2^ or more) [50]. Occupations of the participants were coded using the 3 category National Statistics Socio-economic Classification (NS-SEC) system [51].

Simple thematic analysis was used for the open ended questions by coding the data after familiarisation, and deriving categories and themes inductively.

## Results

### Respondent characteristics

There were 797 completed surveys. Nineteen responses were removed; eighteen repeat responses and one with implausible demographic responses. A total of 778 responses were coded and taken forward for analysis. The participants completing the survey ranged in age from 19 to 62 (mean 33.3, SD 5.7). Most of the respondents (94.8%) were living in the UK, 2.9% were in North America and a small proportion were living in other European countries or the continents of Asia, Australia and Oceania or South America (2.1%). Further analysis was limited to the 688 participants who were living in the UK, of childbearing age (16–44) and who had given birth to at least one child. Characteristics of the participants are presented in Table 1.

**Table 1:** Characteristics of UK respondents compared to UK national values

	UK participants at time of survey (*n* = 688)	National values %
	*n* (%)	
Age	(*n* = 688)	Women giving birth in England
Under 20	2(0.3%)	4.6^a^
20–24	27 (3.9%)	18.2^a^
25–29	140 (20.4%)	28.1^a^
30–34	266 (38.7%)	29.7^a^
35–39	197 (28.6%)	15.5^a^
40+	56 (8.1%)	3.9^a^
Ethnicity	(*n* = 683)	
White	652 (95.5%)	86.0^b^
Black	7 (1.0%)	3.3^b^
Asian	8 (1.2%)	7.5^b^
Mixed	16 (2.3%)	2.2^b^
BMI	(*n* = 663)	All women in England
< 25	286 (43.2%)	42.8^c^
Overweight	213 (32.1%)	33.4^c^
Obese	164 (24.7%)	23.8^c^
Occupation	(*n* = 683)	Female UK residents aged 16–74
Higher managerial, administrative and professional occupations	357 (52.3)	29.0^d^
Intermediate occupations	117 (17.1)	24.0^d^
Routine and manual occupations	59 (8.6)	31.0^d^
Long-term unemployed or never worked	1 (0.1)	6.0^d^
Not classified	149 (21.9)	–
Number of children birthed	(*n* = 681)	
1	322 (46.8)	
2	259 (37.6)	
3 or more	107 (15.6)	

^a^Age at delivery [53]; ^b^ [54]; ^c^ [55]; ^d^ [56]

Most of the participants (95.5%) identified their ethnicity as White, 2.3% as Mixed ethnicity, 1.0% as Black, 1.2% as Asian. The wide geographical distribution of UK respondents can be seen in Additional file 1. The BMI of the respondents ranged from 16.7 kg/m^2^ to 66.6 kg/m^2^ (mean 26.8 kg/m^2^, SD 6.0), 43.2% had a BMI of less than 25 kg/m^2^, 32.1% were categorised as overweight and 24.7% were in the obese category.

The largest occupational group category amongst the participants was higher managerial, administrative and professional occupations (52.3%), although 21.9% of participants’ occupations fell into the NS-SEC unclassified category. A total of 46.8% of the participants had given birth to one child, 37.6% had given birth to two children and 15.6% three or more children.

### Breastfeeding

A total of 677 participants had breastfed (98.4%), with 337 of these women (50.0%) mentioning that they were currently breastfeeding at the time of completing the survey. Only 650 of the participants responded to the question about longest length of time breastfeeding a child, which ranged from 0.05 months to 72 months (mean 17.3, median 15.0 (IQR = 7.7–24.0)); 95.2% reported that they were still breastfeeding at 8 weeks, 84.0% were breastfeeding at 6 months, 64.8% were breastfeeding at 12 months and 58.8% breastfed beyond 12 months.

### Antenatal breast expression - awareness and experience

**Fig. 1:**
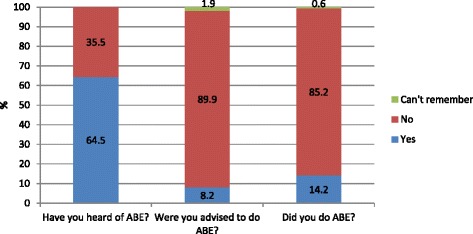
Whether participants had heard of, were advised to do and if they did Antenatal Breast Expression (*n* = 688)

A total of 442 (64.5%) of the respondents had heard of ABE, 56 (8.2%) reported that they had been advised to express breastmilk during pregnancy, and 97 (14.2%) reported that they had undertaken breast expression during pregnancy (See Fig. 1).

**Fig. 2:**
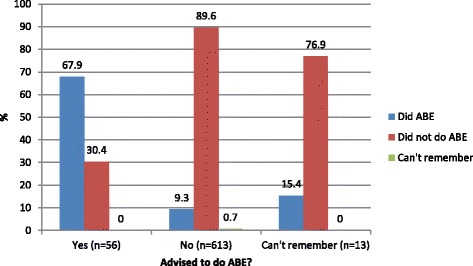
Whether participants who were advised to do ABE undertook ABE (*n* = 682)

Of the 97 participants who had expressed breastmilk during pregnancy, 38 had been advised to (39.2%), 57 (58.8%) had not been advised to and 2 (2.1%) could not remember if they had been advised to. Of the 56 women who reported that they had been advised to express breastmilk during pregnancy, 38 (67.9%) actually expressed, compared to 9.3% of women who had not been advised to express (Fig. 2).

Women who undertook ABE commenced expressing between 0 and 41 weeks of pregnancy, with a median of 36 weeks: 2 (2.2%) reported commencing ABE at 0 weeks of pregnancy, 5 (5.4%) between week 12 and 28, 43 (46.7%) between weeks 28 and 37, and 44 (45.7%) from 37 weeks onwards. Women who had practiced ABE reported giving birth at a mean of 39.7 weeks gestation in their last pregnancy, which was similar to the 39.3 weeks mean length of gestation reported by women who did not undertake ABE.

### Opinions on antenatal breast expression

A majority of respondents, 398 (58.6%), were not sure if ABE was a good idea, with 233 (34.3%) of the respondents stating that they thought ABE was a good idea and 48 (7.1%) thinking ABE was not a good idea (See Table 2). Previous practice of ABE was statistically significantly associated with opinion of ABE (*p* < 0.001); the proportion of women who thought ABE was a good idea was highest in those who had undertaken ABE previously (78.4%), although 26.8% of those who had not previously undertaken ABE still thought ABE was a good idea.

**Table 2:** Participants’ opinions on ABE

		All participants	Previously undertaken ABE?				BMI			
			Yes	No	Can’t remember	*P* value	Under 25 kg/m^2^	Overweight	Obese	*P* value
		*n* (%)	*n* (%)	*n* (%)	*n* (%)		*n* (%)	*n* (%)	*n* (%)	
Is ABE a good idea?	Yes	233 (34.3)	76 (78.4)	153 (26.8)	1 (25.0)	†*p* < 0.001	85 (30.2)	67 (31.8)	76 (46.6)	0.007
	No	48 (7.1)	1 (1.0)	47 (8.2)	0 (0.0)		19 (6.8)	15 (7.1)	11 (6.8)	
	Not sure	398 (58.6)	20 (20.6)	372 (65.0)	3 (75.0)		177 (63.0)	129 (61.1)	76 (46.6)	
Would you consider doing ABE if it was found to help prepare for breastfeeding?	Yes	547 (80.9)	94 (96.9)	445 (78.1)	4 (100)	†*p* < 0.001	220 (79.4)	175 (82.6)	134 (82.2)	0.280
	No	43 (6.4)	1 (1.0)	42 (7.4)	0 (0.0)		15 (5.4)	13 (6.1)	14 (8.6)	
	Not sure	86 (12.7)	2 (2.1)	83 (14.5)	0 (0.0)		42 (15.2)	24 (11.3)	15 (9.2)	

^†^Fisher Exact used as expected count < 5 in over 20% of cells

A total of 547 (80.9%) of participants stated that they would consider doing ABE if it was found to help prepare for breastfeeding (See Table 2). Previous practice of ABE was significantly associated with whether participants would consider doing ABE in the future (*p* < 0.001), with only one participant (1.0%) who had previously done ABE reporting she would not consider doing ABE again if it was found to be helpful to prepare for breastfeeding.

### Overweight and obese subgroups

**Fig. 3:**
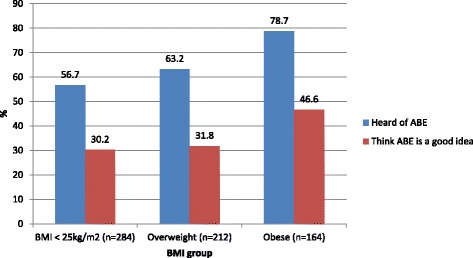
BMI group and whether participants had heard of ABE and opinion on ABE

In this sample, the proportion of women who had heard of ABE increased with increasing BMI group; 56.7% of women with a BMI of less than 25 kg/m^2^ had heard of ABE compared to 63.2% of participants with a BMI within the overweight subgroup 78.7% within the obese subgroup (See Fig. 3). This association showed statistical significance (*p* < 0.001).

The proportion of women who thought ABE was a good idea also increased with increasing BMI group (See Fig. 3); 46.6% of obese participants thought it was a good idea, compared with 31.8% of those who were overweight and 30.2% of those with a BMI of less than 25 kg/m^2^, and this association was statistically significant (*p* = 0.007). However, there was no significant association between BMI group and whether participants would consider doing ABE in the future (*p* = 0.280).

### Qualitative data - Women’s opinions on antenatal breast expression

Several themes emerged from the qualitative data (See Table 3). Amongst participants who felt ABE was a good idea these were; beneficial when mother or baby have medical problems, and preparation for breastfeeding. The themes that emerged amongst participants who thought that ABE was not a good idea were; interfering with nature and harmful. For those who were unsure if ABE was a good idea the main theme was lack of knowledge.

**Table 3:** Themes from the qualitative data

Participant opinion on ABE	Themes from qualitative data	
	Second order themes	First order themes
It’s a good idea	Beneficial when mother or baby have medical problems	Helpful if mother: has gestational diabetes has pre-existing diabetes had breast surgery has low supply is unwell has complications after birth
		Beneficial if baby: has low blood sugar has to go to neonatal unit is premature has jaundice has weight loss has difficulty feeding has tongue-tie
	Preparation for successful breastfeeding	Gaining confidence with expressing technique Establishing a supply of milk Hormone stimulation Encourage milk production and supply Avoid the use of formula milk
		To promote labour
It’s not a good idea	Interfering with nature	Nature gets it right Milk extraction should be after birth Using up the colostrum No need to interfere
	Harmful	May induce early labour or miscarriage Painful Stressful Bullying women to do it
I’m not sure if it’s a good idea	Lack of knowledge	Never heard of it Don’t know of risks/benefits

### Positive perceptions

Participants who thought that ABE was a good idea felt it would be beneficial where mother or baby have medical problems including gestational or pre-existing diabetes, previous breast surgery, complications after birth, prematurity, low blood sugars, admission to neonatal unit or difficulties feeding;

*"If there are any complications during labour which meant that you were unable to feed initially (I.e. PPH [postpartum haemorrhage], or surgery was required taking you away from baby) baby could be spoon or cup feed colostrum. Equally if baby is struggling with blood sugars, jaundice, weight loss."*

*"I have type 1 diabetes, antenatal expression enabled me to collect colostrum and give it to my babies at birth preventing them from having low blood sugar."*

They also referred to ABE as positive preparation for successful breastfeeding, including the opportunity to become more confident with the expressing technique that they could use again after the birth, establishing a supply of colostrum, encouraging the milk supply and avoiding the use of formula milk;

*"A good idea to have some milk stored to avoid formula top ups if struggling to feed."*

*"I would have found it helpful…to have already got used to hand expressing as this was something I needed to do a lot once baby was born."*

A few women also reported that ABE could be beneficial in promoting the onset of spontaneous labour;

*"…It is used to induce labour naturally."*

### Negative perceptions

Participants who felt that ABE was a bad idea raised concerns that it would be harmful; causing preterm labour, painful to undertake, stressful, and result from bullying women to do it. They were also concerned that it was interfering with the natural process of initiating breastfeeding;

*"I worried it would cause early labour."*

*"Nature gets this right, no need to interfere."*

*"Worried of making that [colostrum] go and turn straight into milk when baby arrives."*

### Uncertain perceptions

The predominant theme amongst the participants who were unsure if ABE was a good idea was their lack of knowledge; either that they had not heard of it or did not have enough information about the benefits or risks involved;

*"Have never heard of it or its benefits /negatives."*

*"I didn't know it could be done."*

*"I…was never given any information."*

The participants identified a number of factors that would encourage them to express breastmilk in pregnancy (See Table 4).

**Table 4:** Factors that would encourage participants to express breastmilk in pregnancy

Having evidence-based information about ABE
If ABE was found to increase the likelihood of successful breastfeeding and avoiding the use of formula milk
If there were benefits to the baby
Any medical problems prior to the birth, such as diabetes
Knowing that the baby would have medical problems after birth
Reassurance of the safety of doing ABE
The support of midwives in the antenatal period
The provision of equipment to undertake ABE

## Discussion

This cross-sectional study has provided insight into women’s knowledge and practices surrounding ABE and the acceptability of ABE to mothers. These findings are important as implementation evidence, as this practice is already advised by midwives in many hospitals [30–43], and promoted in some lactation support literature [29, 45].

This survey demonstrated relatively high awareness, with more than half of the participants having heard of ABE. It also found relatively high compliance amongst women who had been advised to do ABE, with 67.9% of women reporting they had followed this advice. Soltani and Scott [49] found lower compliance, with less than half of women who had been advised to express actually undertaking ABE. It is not clear why 32.1% of participants who had been advised to do ABE did not follow this advice and this warrants further investigation into the decision making process for undertaking ABE. Furthermore 20.6% of those who had undertaken ABE were unsure if ABE was a good idea, and further investigation into women’s sources of knowledge about ABE is needed.

Although a majority of participants (57.8%) were unsure if ABE was a good idea, ABE was found to be largely acceptable to the women. A high proportion would consider doing ABE if it was found to beneficial (79.5%), including 96.9% of women who had previously done ABE and 78.1% of those who had not previously undertaken ABE. This reflects wider evidence which demonstrated that 95% of participants who had undertaken ABE would do it again if it was found to be safe and effective [46].

Participants identified ABE as a form of preparation for breastfeeding and helpful in avoiding the use of formula milk. This reflects other findings that women who undertake ABE report increased confidence and readiness for breastfeeding, the benefit of learning the technique to use postnatally, and a reduced need for artificial milk supplementation [46].

Concerns raised by participants about the potential of ABE to cause harm included the pain of the procedure and the risk of causing preterm labour. Forster et al. [46] reported 19.2% of women undertaking ABE experienced nipple pain and 26% experienced Braxton Hicks or contractions, although none attributed ABE to the onset of spontaneous labour. This survey did not enable us to determine if ABE had any impact on timing of the onset of labour. While a large multi-centred randomised controlled trial of women with diabetes in pregnancy who were at low risk of complications suggested no impact on gestational age at delivery from the practice of ABE [48], other smaller studies with less stringent eligibility criteria have suggested both a trend towards lower gestational age at delivery and an increased rate of special care baby admissions for babies whose mothers had undertaken ABE [46, 49]. Further evidence about the safety of ABE is therefore needed, particularly in pregnant women without diabetes. It is notable that a large proportion (54.3%) of women in this survey who undertook ABE commenced ABE prior to 37 weeks of pregnancy, and it is important to inform women if they decide to undertake ABE that commencing after 37 weeks of pregnancy will reduce the likelihood of preterm birth [49].

Women of childbearing age in this survey who were in the overweight or obese subgroups were more likely to have heard of ABE and were more likely to think that ABE was a good idea, and were no less likely, than women of normal weight, to undertaken it if it was found to be helpful for breastfeeding. This is significant as women in the overweight or obese subgroups typically have higher rates of medical problems such as diabetes [6, 23] and lower rates of successful breastfeeding outcomes [6–13]. If the safety and efficacy of ABE is established, they could therefore be considered to be an important target group for this intervention.

### Strengths and limitations

This survey included a large sample of women from a wide spread of UK locations. Comparing UK participants’ characteristics with national population data from England, demonstrated that this sample were more predominantly of a white ethnic group and there were a considerably lower proportion of Asian respondents. The sample was also older and of higher socioeconomic status, as indicated by occupation, than the current childbearing population, and hence some of their views and experiences may not be representative. The BMI distribution of the UK participants was very similar to that in the national population.

Undertaking this survey using online technology facilitated wide access and a high response rate that covered wide geographical areas with minimum resources. A maternity user group representative leading survey recruitment may have been an advantage to encourage such a large response rate. However, as a retrospective, self-reported questionnaire, it may have been subject to selective recall bias. The sample demonstrated a much higher breastfeeding rate than that of the national childbearing population in England; 98.4% of the participants reported they had breastfed, compared with the national breastfeeding rate of 74.3% at birth [52]. The participants had also breastfed for longer with 95.2% breastfeeding at 8 weeks compared to the national figure for England of 43.8% [52], and 84.0% reported breastfeeding at 6 months, compared to 34% of the national childbearing population [5]. This was therefore a self-selected sample of women who were highly motivated and successful breastfeeding mothers, and may not represent the opinions of the wider population. Nevertheless, even assuming a higher rate of motivation, a large proportion had concerns for implementing ABE and in depth analysis of their views are worthy of further investigation and consideration.

## Conclusion

Amongst women who have breastfed, many have heard of ABE, and compliance with advice to undertake ABE is relatively high. ABE appears to be acceptable to many women, including those in overweight or obese subgroups. However, the benefit and safety of ABE needs to be established to address the needs of childbearing women for evidence-based information about this practice. If ABE is demonstrated to be beneficial in the promotion of breastfeeding, overweight and obese pregnant women could be an important target group for this intervention.

## Additional file

Additional file 1: Map showing geographical distribution of UK respondents (*n* = 688). (DOCX 432 kb)

## Abbreviations

ABE: Antenatal breast expression
BMI: Body mass index
IQR: Interquartile range
NS-SEC: National Statistics Socio-economic Classification
PPH: Postpartum haemorrhage
SD: Standard deviation
UK: United Kingdom
