# Supplementary figures and images for: Women’s perspectives on antenatal breast expression: a cross-sectional survey
Source: Reprod Health. 2018 Apr 4;15:58. doi: 10.1186/s12978-018-0497-4 (PMC5885364; doi:10.1186/s12978-018-0497-4)

**
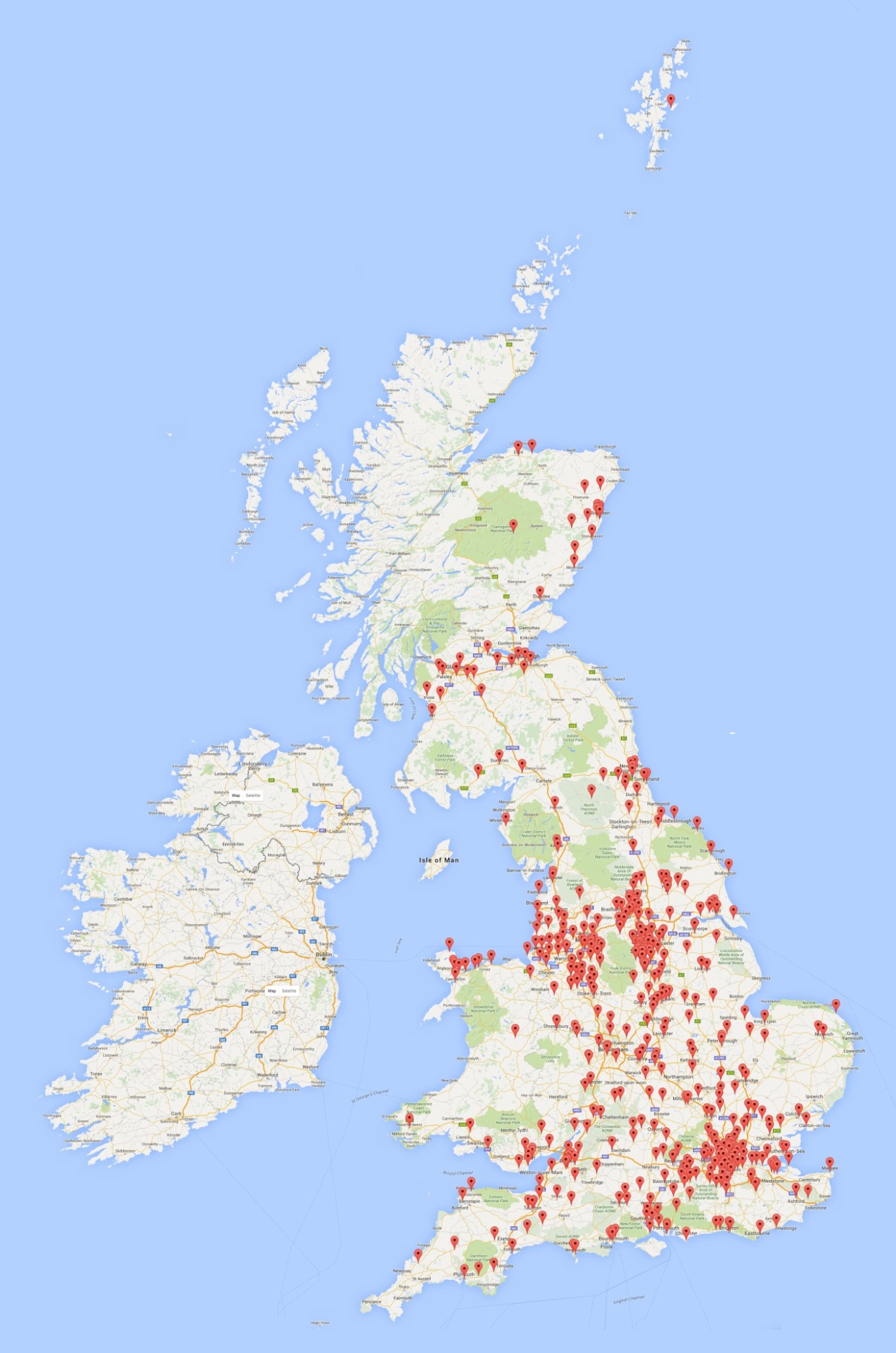
Additional file 1 Map showing geographical distribution of UK respondents (n=688)**

Supplement: Supplementary file 1 — Map showing geographical distribution of UK respondents (n = 688). (DOCX 432 kb) [file 12978_2018_497_MOESM1_ESM.docx]
